# Recent advances in the synthesis and application of biomolecular condensates

**DOI:** 10.1016/j.jbc.2025.108188

**Published:** 2025-01-13

**Authors:** Zhongyue Li, Wei Tan, Guo-ping Zhao, Xiangze Zeng, Wei Zhao

**Affiliations:** 1 CAS Key Laboratory of Quantitative Engineering Biology, Shenzhen Institute of Synthetic Biology, Shenzhen Institutes of Advanced Technology, Chinese Academy of Sciences, Shenzhen, China; 2 CAS Key Laboratory of Synthetic Biology, CAS Center for Excellence in Molecular Plant Sciences, Shanghai Institute of Plant Physiology and Ecology, Chinese Academy of Sciences, Shanghai, China; 3 State Key Lab of Genetic Engineering & Institutes of Biomedical Sciences, Department of Microbiology and Microbial Engineering, School of Life Sciences, Fudan University, Shanghai, China; 4 Department of Physics, Hong Kong Baptist University, Kowloon Tong, Hong Kong

**Keywords:** biomolecular condensates, phase separation, synthetic biology, SBMCs, *de novo* synthesis

## Abstract

Biomolecular condensates (BMCs) represent a group of organized and programmed systems that participate in gene transcription, chromosome organization, cell division, tumorigenesis, and aging. However, the understanding of BMCs in terms of internal organizations and external regulations remains at an early stage. Recently, novel approaches such as synthetic biology have been used for *de novo* synthesis of BMCs. These synthesized BMCs (SBMCs) driven by phase separation adeptly resemble the self-assembly and dynamics of natural BMCs, offering vast potentials in basic and applied research. This review introduces recent progresses in phase separation–induced SBMCs, attempting to elaborate on the intrinsic principles and regulatory methodologies used to construct SBMCs. Furthermore, the scientific applications of SBMCs are illustrated, as indicated by the studies of chromosome structure, pathogenesis, biomanufacturing, artificial cell design, and drug delivery. The controllable SBMCs offer a powerful tool for understanding metabolic regulations, cellular organizations, and disease-associated protein aggregations, raising both opportunities and challenges in the future of biomaterial, biotechnology, and biomedicine.

Cells execute programmed processes by organizing the intracellular space into small substructures, known as organelles or compartments (1). While eukaryotic cells are characterized by membrane-bound organelles, both prokaryotic and eukaryotic cells encompass a group of membraneless organelles named biomolecular condensates (BMCs). These BMCs, including but not limited to nucleoli, Cajal bodies, stress granules, processing bodies, and germ granules, are devoid of lipid membranes, presenting unique biochemical milieu and organizational properties (2, 3). Phase separation represents a thermodynamic phenomenon in which a homogenous mixture spontaneously separates into two or more distinct phases, each with a distinct concentration of components (4). As a burgeoning concept in cell biology, phase separation is instrumental in driving the formation of fluidic condensed BMCs by clustering proteins and/or nucleic acids, shaping the spatial and temporal dynamics in cells. The phase separation–driven BMCs are integral to several biological processes, including gene transcription (5, 6), chromatin organization (7), DNA damage (8), cell division (9, 10), tumorigenesis (11), carcinogenesis (12), and aging (13).

While prior studies focus on the identification and functional analysis of natural BMCs, increasing research on the synthesis and regulation of BMCs has been conducted. These synthesized BMCs (SBMCs) mimic the dynamics of natural BMCs and hold promise in diverse applications, such as cellular architecture study (14, 15), biomanufacturing engineering (16), drug delivery (17), and disease treatments (18, 19). Despite these advances, challenges remain in the *de novo* synthesis of functional and controllable SBMCs, particularly in living cells.

In this article, we comprehensively reviewed recent advances in crafting and modulating SBMCs through phase separation. The basic principles used for constructing SBMCs and the key methodologies in the regulation of SBMCs were summarized. Furthermore, the applications of these controllable SBMCs were demonstrated, and representative examples were highlighted. We also outlined the challenges and prospects of SBMCs, spotlighting potential trajectories for future inquiry and innovation. Collectively, this review assists in understanding the reconstitution and engineering of BMCs and provides perspectives on applications and challenges of SBMCs, particularly for those of metabolic regulations, cellular organization, and neurodegenerative disease treatments.

## Basic principles of phase separation

### Multivalent motifs in phase-separating proteins

In biological systems, molecular multivalency is the primary driving force for phase separation and BMC assembly (2, 20). The characteristic multivalent motifs in phase-separating proteins include intrinsically disordered regions (IDRs), DNA-recognition motifs/RNA-recognition motifs (RRMs), multiple folded domains (MFDs), and prion-related domains (21, 22). For instance, BMCs like processing bodies and promyelocytic leukemia nuclear bodies are rich in IDRs and RRMs, respectively (23, 24). Among these motifs, IDRs lacking stable folded structures play a central role in phase-separating proteins. DNA-recognition motifs/RRMs are a widespread class of DNA-/RNA-binding motifs that participate in nucleotide-induced phase separation, *via* specific recognition of nucleotide sequences (25). On the other hand, MFDs are able to provide multiple interaction interfaces, facilitating the formation of complex protein–protein interaction networks (26). These structural characteristics enable proteins to form dynamic and reversible BMCs and perform essential physiological functions in cells.

### Multivalent interactions drive protein phase separation

**Figure 1:**
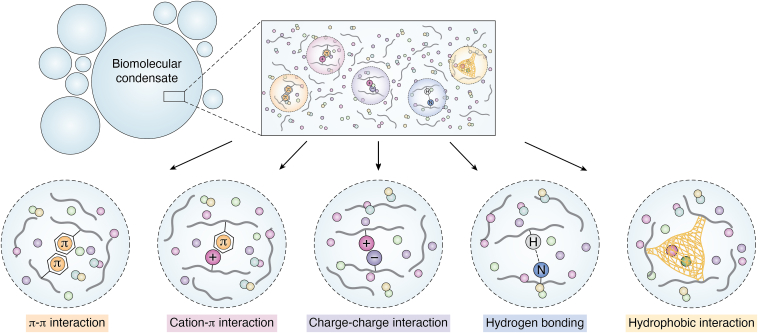
**Schematic of phase separation driven by multivalent interactions.** The multivalent interactions, including π–π, cation–π, charge–charge, hydrogen bonding, and hydrophobic, operate independently or synergistically to drive phase separation. These multivalent interactions facilitate the formation of dynamic biomolecular condensates.

Multivalent interactions in phase-separating proteins effectively manage the energy requirements for biomolecule separation (27). These interactions offset the entropy costs by fostering a network of synergistic, transient, and dynamic molecular associations, allowing proteins to attain various conformations and engage with diverse binding partners (2) (Fig. 1).

The π–π interaction involving the planar configuration of sp^2^ hybrid atoms is one of the most common multivalent interactions (2, 28, 29). These sp^2^ hybrid atoms are prevalent in aromatic amino acids, such as Tyr, Trp, and Phe. Also, the sp^2^ hybridizations occur in carboxyl or carboxyl amine groups of Gln, Asn, Asp, and Glu as well as in the guanidine groups of Arg (28, 30). Alongside π–π interactions, the roles of cation–π interaction as driving forces for protein phase separation are increasingly recognized (31). The cation–π interaction typically occurs between the positively charged amino acids and aromatic amino acids. Studies on the RNA-binding protein FUS and human nuclear ribonucleoprotein A1 (hnRNPA1) highlight the significance of these interactions in phase separation, as demonstrated by the suppression of phase separation upon mutation of aromatic amino acids (32, 33). Recently, the strengths of these interactions have been quantified from different levels of calculations (4, 34, 35).

Charge–charge interaction drives phase separation in some cases. Proteins or polymers with opposing charges tend to aggregate upon proximity, forming liquid-like structures *via* charge neutralization. Notably, the fully intrinsically disordered proteins (IDPs) often exhibit a high abundance of charged amino acids (36). The interactions between charged side chains of these amino acids enhance the occurrence of protein phase separation (37).

Hydrogen bonding, known for its dynamic and transient nature, is vital in the stability of BMCs. Most amino acids, especially polar and charged representatives, can form hydrogen bonds. These bonds contribute significantly to the stabilization of protein core network through inter-residue interactions. For instance, the high density of polar side chains in the fibril core of human FUS low-complexity protein (FUS-LC) suggests a substantial role in forming hydrogen bond networks, necessary for the stability of its BMC (34, 38).

Hydrophobic interaction is involved in the phase separation of proteins with abundant hydrophobic amino acids. Amino acids like Leu, Ile, and Val tend to cluster in specific regions. These hydrophobic interactions bolster the hydrogen bond interactions of intermolecular backbones and foster cross-β structure formation (39). Elbaum–Garfinkle group found that changing the length of the hydrophobic domain at the end of minielastin can modulate its maturation process, providing mechanistic insights into the formation and maturation of the elastin-like BMCs (40). Tuomas Knowles group demonstrated that proteins like FUS, TDP-43, and Sox2 could re-enter BMC states under high salt concentrations by hydrophobic and nonionic interactions. This re-entrance into phase separation is unlike the low-salt phase separation, where the condensates are stabilized by electrostatic forces (34).

### Sequence features of the multivalent motifs

As outlined previously, proteins with multivalent motifs have the potential to undergo phase separation. IDRs featuring intrinsically disordered sequences enable the containing proteins exhibiting a high degree of conformational flexibility. Gly is one of the most frequent residues in IDRs undergoing phase separation, particularly in RGG motifs. The representative proteins containing RGG motifs include FUS, Ewing's sarcoma protein, and TATA-binding associated factor 15 (41, 42). The hydrophilic and charged residues (such as Pro, Ser, Glu, Arg, and Lys) are also commonly found in IDR sequences, and they usually engage in dynamic interactions and promote disordered states. Proteomic analysis highlights the prevalence of the P-X_n_-G motif in IDRs, where “X” represents any amino acid and “n” varies from 0 to 4. The P-X_n_-G motif is considered as a structural scaffold for the encoded BMCs (43). In addition, Gln and Asn are prevalent in a group of prion-like IDR-containing proteins, such as TDP-43 and hnRNPA1 (44, 45). It was found that the protein phase behavior is correlated with the length of the IDR chain. At a given temperature, the saturation concentration (C*t*) will be lower for proteins with longer IDR lengths (46). The results align with the classic Flory–Huggins theory, linking sequence features to the functional propensities of phase-separating proteins (47, 48, 49).

Stickers and spacers model is proposed by Pappu *et al.* (50) and then further validated by experiments by Tanja Mittag group (29). This model involves aromatic residues like Phe and Tyr acting as stickers and polar residues and glycine serving as spacers, which combine to determine the overall phase behavior of IDPs. The valence (number) of stickers regulates the extent of molecular compaction, whereas the pattern (relative positions along the sequence) of stickers determines the ability of IDPs to undergo phase separation *versus* aggregation. Using the stickers and spacers model, Derek Woolfson group were able to synthesize a series of SBMCs in *Escherichia coli*, with altered numbers of aromatic residues in the α helical repeats and varying lengths and polarity in the linkers (14).

The representative feature of MFDs is the presence of repetitive sequences with alternating net charges, typically 8 to 10 residues in length (51). These sequences are rich in Phe/Arg/Gly motifs (*e.g.*, FG, GF, GR, RG) and are often found within positively charged areas. For instance, the phase separation of the nuclear pore complex Nup98 relies on repetitive FG motifs, associated with chromosomal translocation in African *Xenopus* (52). In addition, heterologous expression of hnRNPA1 produces reversible amyloid-like structures in *E. coli*, using repetitive motifs like GFGGNDNFG, GFGNDGSNF, or YNDFGNY (53). Another significant sequence feature of MFDs is the ability to interact with short linear motifs, promoting phase separation (54). For instance, there is an enhanced phase separation for the repetitive proline-rich motif (PRM)_n_ after interacting with the linear SRC homology 3 (SH_3_)_n_ domain (20).

Hence, the different sequence features have been shown for different multivalent motifs. It is worth noting that there is no “one-fit-all” features for all multivalent motifs. Moreover, because of the limited training data (or available validated sequences), it is still very challenging to predict whether a given sequence could phase separate or not.

## Regulatory methods for SBMCs

The presence of featured sequences does not imply the inevitability of protein phase separation, as the formation of BMCs is also regulated by cellular environments and external stimuli, such as protein concentration, temperature, pH, ionic strength, and osmotic pressure. To achieve SBMCs, rational design to mimic the components of natural BMCs and comprehensive engineering to modulate external factors are necessary (Table 1). Several representative examples are reviewed later.

**Table 1:** Representative examples and applications of constructing SBMCs

Name	Multivalent motifs	Regulatory motifs	*In vivo*/*in vitro*	Applications	Reference
Light-controlled phase separation					
OptoDroplet	IDR	Cry2	Mammalian cells	Studying protein phase separation dynamics in live cells	(58)
OptoDropletTFs	IDR	Cry2-CIBn	Mammalian cells	Controlling localization and activity of transcription factors with light	(6)
Corelets	IDR	SspB–iLID	Mammalian cells, yeast, *Caenorhabditis elegans*	Studying protein interactions and mapping intracellular phase diagrams	(62)
PixELLs	IDR	PixE–PixD	Mammalian cells	Studying protein phase separation dynamics in live cells, especially for the condensate disassembly	(63)
CasDrop	IDR	SspB–iLID	Mammalian cells	Exploring the nuclear chromatin restructuring	(65)
iPolymer-Li	MFD	SspB–iLID	Mammalian cells	Creating reversible protein-based hydrogels	(70)
-	N-terminal IDR from FUS	Cry2, Cry2olig, PixE-PixD	*Saccharomyces cerevisiae*	Increasing product formation and specificity by light-switchable clustering	(118)
Binary population of coacervates	DDAB and PDDA	Photoactive *trans*-AzoAsp_2_	*In vitro*	Protocell synthesis	(106)
RMR/WSCP	RGG fused mussel foot protein	WSCP	Mammalian cells	Triggering a liquid-to-solid phase transition	(67)
-	Polylysine	Arylazopyrazole-conjugated ssDNA	*In vitro*	Regulating reactions *via* dynamic LLPS	(119)
Chemical controlled phase separation					
iPolymer	MFD	Rapamycin	Mammalian cells	Creating irreversible protein-based hydrogels	(69)
-	MFD	Rapamycin	*In vitro*	Engineered condensates boost SUMOylation rates	(120)
Metal-induced phase separation	PRM-SH3 with 6xHis tag	Metal ions	*In vitro*	Studying protein phase separation and engineering BMCs	(71)
Biphasic regulator	IDR	Bis-ANS	Yeast	Modulating protein phase separation using small molecules	(72)
Enzyme controlled phase separation					
REPS	IDR and MFD	Protease	Mammalian cells	Achieving enzyme-controlled phase separation	(74)
Temperature-controlled phase separation					
PNIPAM-GO system	PNIPAM	Temperature	*In vitro*	Controllable wastewater treatment	(83)
-	ELPs	Temperature	*In vitro*	Demonstrating biresponsive phase separation in biological contexts	(82)
pH-controlled phase separation					
-	ELPs	pH	*In vitro*	Demonstrating as versatile pH-responsive biomaterials	(87)
-	PAAD and PGA	pH	*In vitro*	The biodegradable and pH-sensitive hydrogels for drug delivery carriers	(90)
Other controlled phase separation					
-	IDR and MFD	RIAD-RIDD	*Escherichia coli* (*E. coli*)	Facilitating enhanced biosynthesis of farnesene	(100)
Spider silk proteins	IDR and MFD	Spontaneously	*E. coli*	Facilitating enhanced biosynthesis of 1,3-diaminopropane	(15)
Artificial IDPs	IDR	Spontaneously	*E. coli*	Controlling cellular functions through phase separation	(121)
Artificial IDPs	IDR	Spontaneously	*S. cerevisiae*	Boosting assimilation of methanol and malate, enhancing chemical productions	(122)
IPH	MFD	Spontaneously	*In vitro*	Enriching biomolecules, enhancing enzymatic reactions	(120)
DNA-encoded artificial cell	LAC	Spontaneously	*In vitro*	As a biosensor that amplifies peroxidase-like activity	(123)
-	Hydrophilic polymer HPMC and hydrophobic polymer PLA	Spontaneously	*In vitro*	Using phase-separated polymer blends to control drug release in oral solid dosage forms	(109)
-	Histones with poly(dA)	Spontaneously	Mammalian cells	Retaining chemotherapeutic agents for controlled drug delivery	(108)

DDAB, didodecyldimethyl ammonium bromide; HPMC, hydroxypropyl methylcellulose; IPH, IDP mimicking polymer-oligopeptide hybrid; LAC, artificial cell with a liquid core; PAAD, polyacrylic acid derivative; PDDA, poly(diallyldimethylammonium chloride); PGA, poly(l-glutamic acid); PLA, polylactic acid; *trans*-AzoAsp_2_, photoactive anionic aspartic-acid-appended azobenzene derivative.

### Light-controlled phase separation

**Figure 2:**
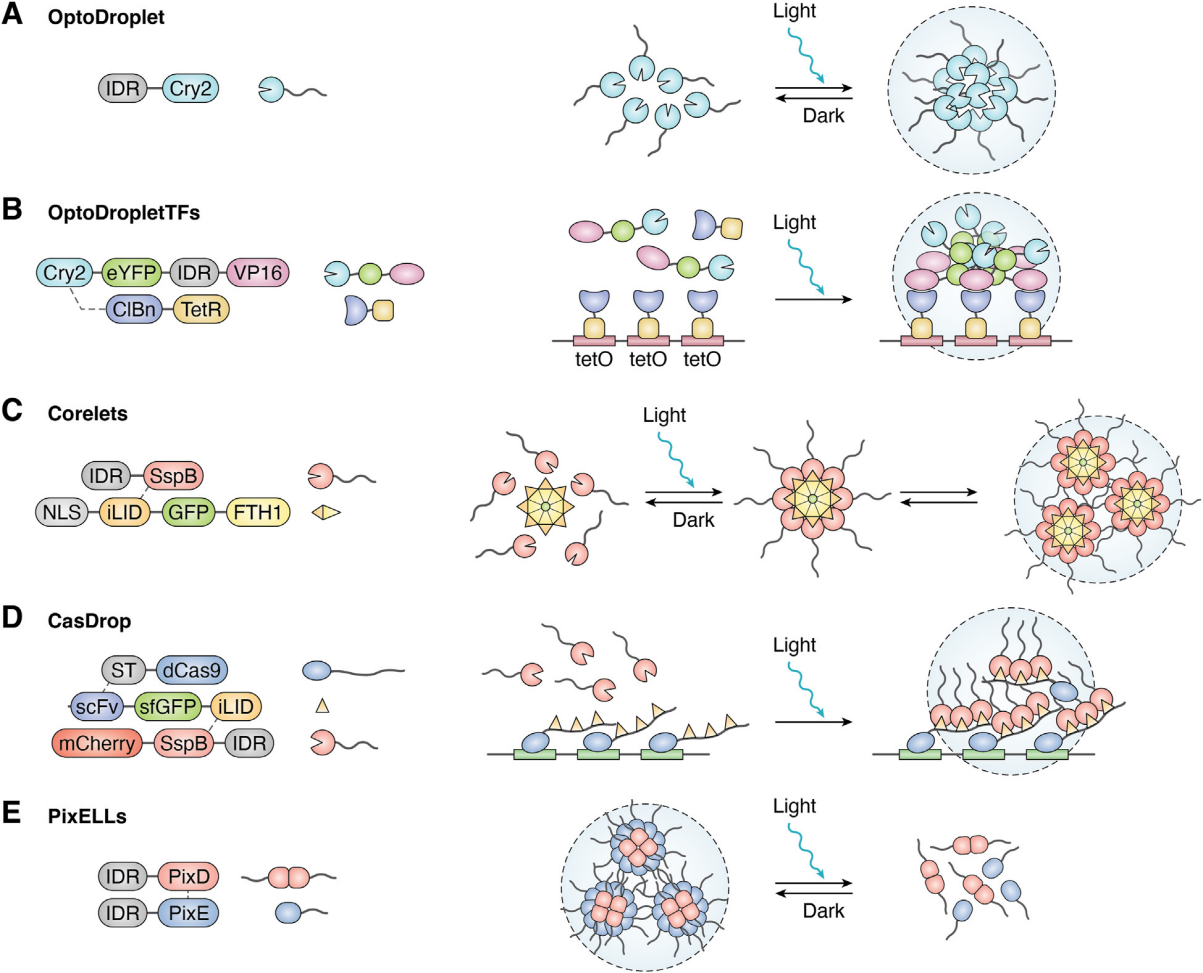
**Schematic of light-controlled phase separation.***A*, OptoDroplet employs the photosensitive protein Cry2 (cryptochrome 2) to regulate biomolecular condensate formation under light exposure. *B*, OptoDropletTFs, specific to the TetR–tetO system, uses light to regulate gene expression by engineering the phase separation of transcription factor TetR. *C*, Corelets utilize FTH1 and the photosensitive proteins iLID–SspB to self-assemble into spherical particles consisting of 24 subunits of IDRs, enabling precise regulation of protein assembly and disassembly. *D*, CasDrop is founded on the CRISPR–Cas system and uses light to specifically control the local assembly of dCas9 complexes. *E*, PixD and PixE in the PixELLs system oligomerize in the dark and dissociate into PixD and PixE monomers upon light stimulation, demonstrating its utility in light-controlled biomolecular condensates. *Dashed lines* indicate the interactions between protein modules. dCas9, dead Cas9; FTH1, ferritin heavy chain 1; IDR, intrinsically disordered region; iLID, improved light-induced dimer protein.

Light-controlled systems have been comprehensively employed for precise modulation of intracellular activity, drug release, and assembly of biomaterials (55, 56, 57). Clifford Brangwynne group reported an OptoDroplet system in 2017, which can induce light-dependent phase separation of proteins (58) (Fig. 2*A*). Fusion of IDRs with the photolytic enzyme homologous region of *Arabidopsis* cryptochrome 2 (Cry2) results in light-responsive clusters. Once the clusters reach a specific concentration, photoactivated phase separation occurs, enabling controllable SBMC production. The system employed IDRs from three RNA-binding proteins: FUS, DDX4, and hnRNPA1, each forming SBMCs upon blue light activation.

Based on OptoDroplet, Wilfried Weber group developed a photo-controlled device for mammalian transgenic expression, called OptoDropletTF (6). It was designed utilizing the Tet system, aiming to attain higher transcriptional activity at lower light doses to overcome limited light penetration in tissues or live animals (Fig. 2*B*). The synthetic transcription factor is composed of Cry2-eYFP-IDR-VP16, in which VP16 is a transactivation domain. It also includes an optical control switch, employing cryptochrome-interacting basic–helix–loop–helix-TetR as the DNA-binding domain. Blue light activates the cryptochrome-interacting basic–helix–loop–helix and Cry2 homologous oligomerization in the presence of cryptochrome, causing the formation of SBMCs. The platform utilizes an optogenetic method to precisely control the condensate formation, significantly enhancing the activity of synthetic transcription factors (59, 60, 61).

In 2018, Clifford Brangwynne group introduced a light-controlled system, Corelets, enabling precise subcellular localization of phase-separating proteins (62) (Fig. 2*C*). The system includes two modules: one GFP-labeled ferritin heavy chain 1, which is fused with a nuclear localization signal and an improved light-induced dimer protein (iLID), and another including IDR fused to a stringent starvation protein B (SspB). Under blue light activation, SspB dimerizes with iLID to generate IDR-containing condensates. These condensates can be captured and assembled by ferritin heavy chain 1, forming spherical droplets through oligomerization and targeting cell nuclei *via* nuclear localization signal.

CasDrop (63) is another optogenetic platform developed by Yongdae Shin *et al.*, aiming to precisely localize nuclear BMCs using dead Cas9 (dCas9) (Fig. 2*D*). The first portion of the system is composed of a dCas9 fused to the SunTag that can target specific genomic sites using guide RNA. The second portion is a single-chain fragment variable antibody (scFv) fused to a superfolder GFP (sfGFP) and an optogenetic dimerized protein iLID, namely scFv–sfGFP–iLID. The scFv antibody can bind to SunTag specifically. The third part of this system is IDR-mCherry-SspB. When stimulated by blue light, the IDR oligomers are formed by the SspB–iLID interaction and could be further localized at specific genomic sites *via* dCas9-based scaffolds. The system facilitates the exploration of chromatin dynamics, shedding light on the impacts of BMCs on nuclear chromatin restructuring (3, 64).

PixELLs, in contrast to OptoDroplets, was designed to leverage light to induce the dissociation of protein condensates (Fig. 2*E*). The proteins PixD and PixE from *Synechocystis* can form BMCs in the dark, setting the basis of PixELLs system developed by Jared Toettcher group (65). The system takes the advantage of rapid dissociation of the PixD–PixE complex under light stimulation, allowing for rapid and localized dissociation of protein condensates. The system's reversibility offers unique insights into the dynamics of protein condensates (58, 66).

Collectively, light-controlled phase separation demonstrated outstanding advances in the construction of SBMCs with high spatiotemporal resolution. The noninvasive and reversible regulation of SBMCs indicate it can be comprehensively used for basic research and other scientific or medical applications. Nevertheless, this approach typically requires continuous light exposure to maintain phase separation, which could introduce practical limitations, including device dependence and phototoxicity (58, 63, 65, 67, 68).

### Chemical- and enzyme-controlled phase separation

Compared with light-controlled phase separation, the chemical- or enzyme-controlled phase separation has advantages when phototoxicity or invalid device happens, making them more practical and reliable in some specific situations.

**Figure 3:**
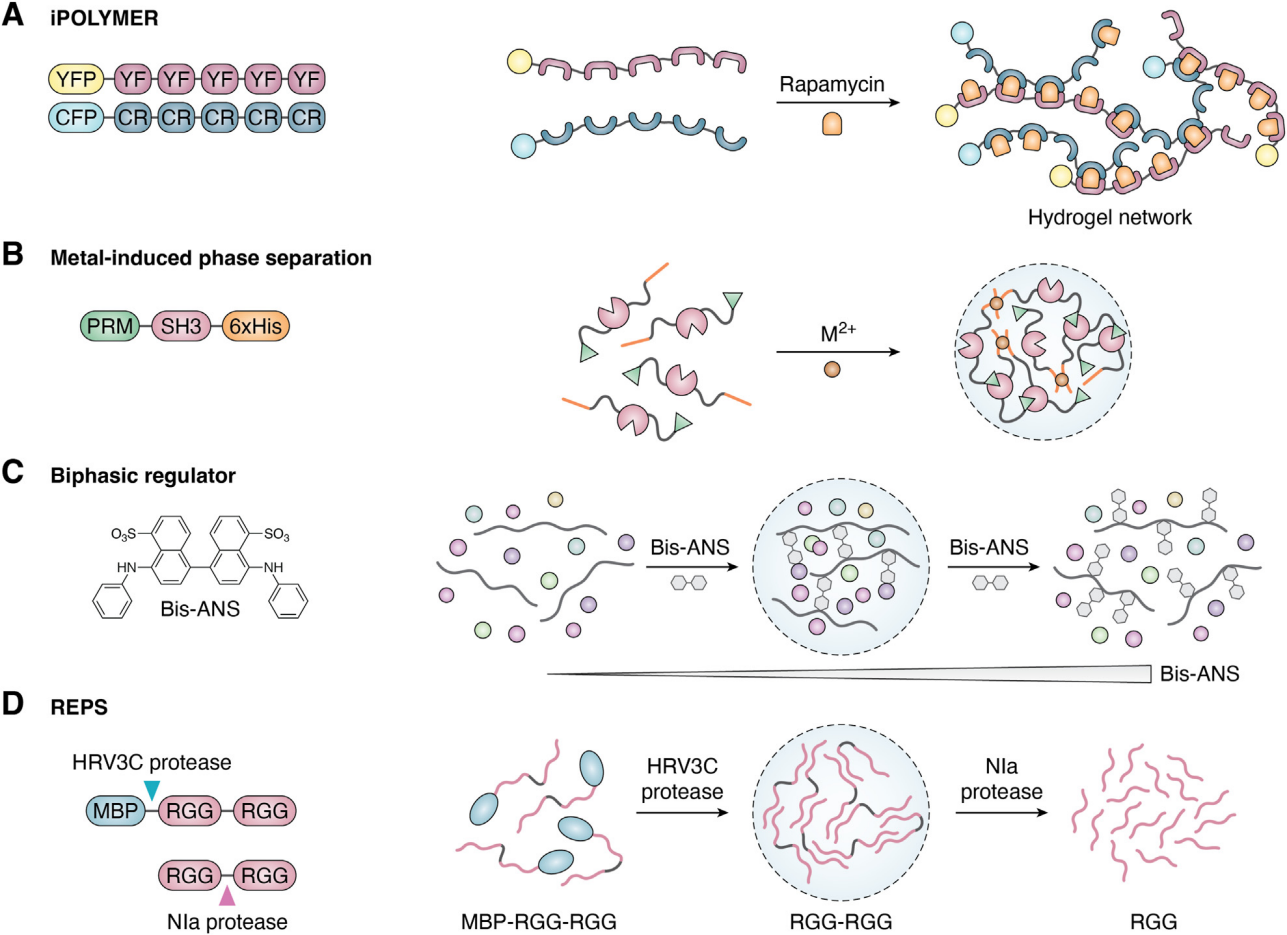
**Schematic of chemical- and enzyme-controlled phase separation.***A*, iPOLYMER is a chemically regulated method for the construction of SBMCs. The dimerization of YFP–FKBP (YF) and CFP–FRBP (CR) induced by rapamycin leads to phase separation. *B*, the interaction between metal ions (*e.g.*, Ni^2+^) and hexahistidine tags (6x-His) induces the fusion protein PRM–SH_3_ to phase separation. *C*, Bis-ANS serves as a biphasic regulator that adjusts the hydrophilic and hydrophobic properties of the system, controlling the occurrence of phase separation. *D*, an RGG-based enzyme-triggered phase separation control system (REPS) uses HRV3C protease to promote BMC assembly by cleaving maltose-binding protein and NIa protease to disassemble BMC by cleaving multiple RGG domains into individual domains. BMC, biomolecular condensate; FKBP, FK506-binding protein; PRM, proline-rich motif; SBMC, synthesized BMC; SH_3_, SRC homology 3.

Takanari Inoue group reported iPOLYMER (69), a technique leveraging rapamycin-induced dimerization to produce protein-based hydrogels in living cells (Fig. 3*A*). By dimerizing FK506-binding protein (FKBP) and FKBP rapamycin-binding protein *via* rapamycin, hydrogels were generated using each five repeats of YFP–FKBP and CFP–FRBP (YF_x5_ and CR_x5_). The fluorescence recovery after photobleaching assay indicated that the hydrogel formation is irreversible. Nevertheless, the newly reported iPOLYMER-LI solves the irreversibility problem, enabling reversible modulation of protein condensates. As a novel construction technique, the iPOLYMER system provides a foundation for the synthesis of functional BMCs (70).

Metal ions alter the affinities of multivalent interactions and influence protein phase separations. Also, metal ions could be used to develop BMCs as they regulate the receptor–ligand pairs in some cases. The fused protein PRM–SH_3_ with a 6x-His tag undergoes phase separation in the presence of NiCl_2_ (71). The introduction of Ni^2+^ increases the system’s turbidity, resulting in SBMC formation. In contrast, the addition of EDTA, a metal ion chelator, disrupts the receptor–ligand pairs and reduces the turbidity of the system (Fig. 3*B*). Together, these findings underscore the role of metal ions in regulating protein phase separation, offering an avenue to understand cellular processes and engineer SBMCs.

Witold Surewicz group have demonstrated that some compounds, notably 4,4′-dianilino-1,1′-binaphthyl-5,5′-disulfonic acid (Bis-ANS), are effective biphasic regulators of protein phase separation (72). Bis-ANS is a fluorescent molecule usually bound to hydrophobic patches of proteins (73). The study revealed that Bis-ANS and analogous compounds effectively induce phase separation of yeast TDP-43 at lower concentrations. However, at relatively higher concentrations, the compounds disrupt BMCs *via* electrostatic repulsion (Fig. 3*C*). This discovery introduces a bidirectional approach to coordinate phase separation using small molecules, laying the foundation for drug developments with therapeutic potential in tuning phase separation.

To achieve a more practicable manipulation of phase separation, Daniel Hammer and Matthew Good group innovated a minimal, enzyme-triggered system called REPS, based on the RGG motif (74) (Fig. 3*D*). The RGG motif is an RNA-binding fragment often found in phase-separating proteins, and experimental results suggest that the tandem RGG domains are more prone to phase separation. In the REPS system, maltose-binding protein (MBP), commonly used as a solubility-enhancing label (75), is fused to the N terminus of RGG–RGG to produce MBP–RGG–RGG. The introduction of HRV3C protease (76) triggers the rapid formation of BMCs by removing the MBP tag. Moreover, a specific sequence, ENLYFQG, was designed between RGG domains, operating as a recognition site for NIa protease (77). Following treatment with NIa protease, the RGG–RGG protein is cleaved into single RGG units, inducing the dissociation of BMCs under physiological conditions and achieving enzyme-controlled phase separation.

In summary, these innovations highlight the power of chemical- and enzyme-triggered systems in producing controllable SBMCs. By utilizing specific molecules or proteases, precise control over the assembly and/or disassociation of BMCs was achieved (72, 74).

### Temperature-controlled phase separation

**Figure 4:**
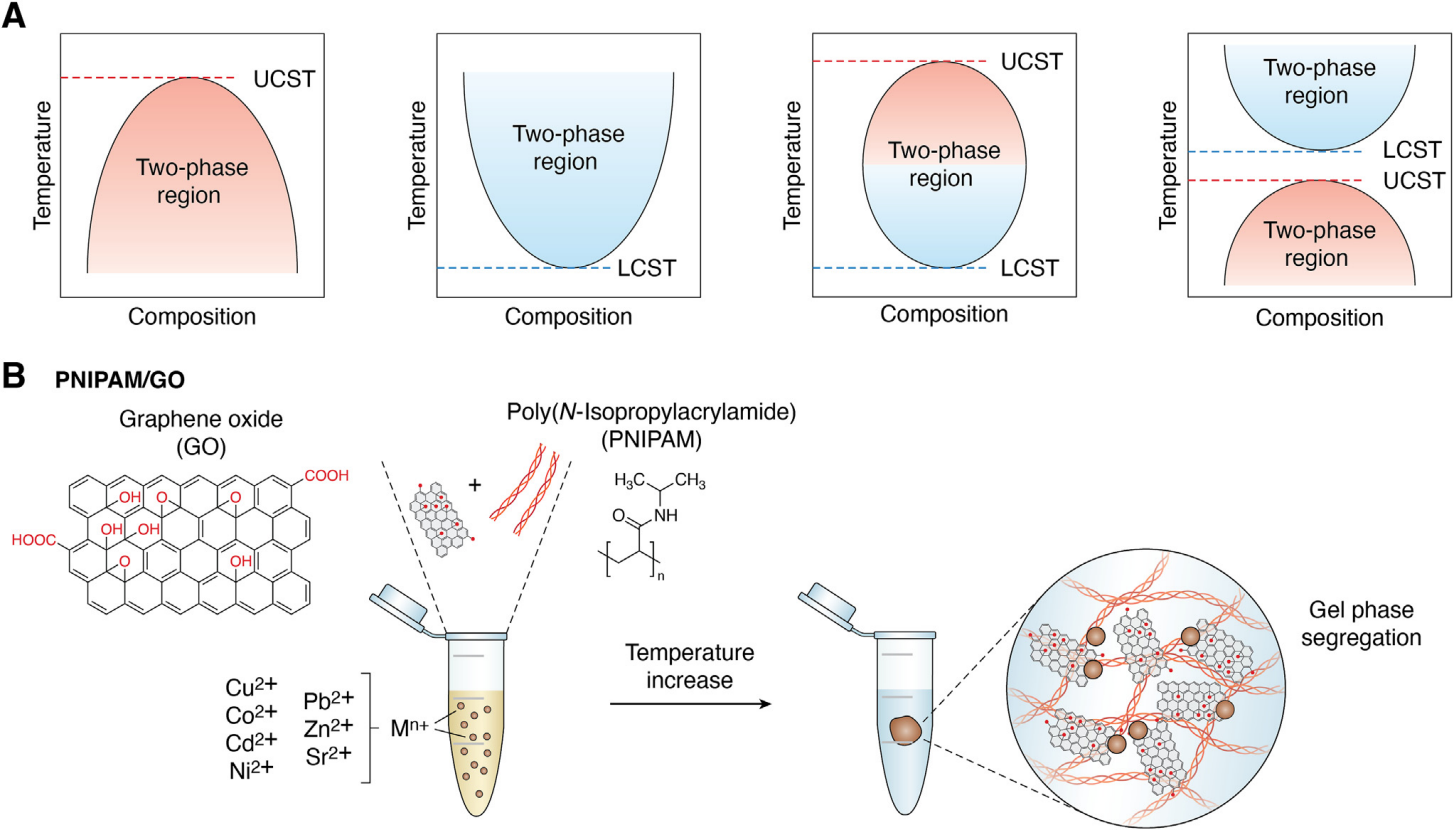
**Schematic of temperature-controlled phase separation.***A*, design of synthetic BMCs using proteins with temperature-sensitive phase behaviors. For proteins with UCST behavior, the liquid phase forms below the critical temperature, whereas for proteins with LCST behavior, the liquid phase is formed above the critical temperature. When utilizing proteins with dual thermoresponsive properties, the system exhibits two-phase behavior within a specific temperature range, bounded by UCST on one end and LCST on the other end. *B*, PNIPAM–GO is a temperature-controlled phase separation system leveraging the adsorption capability of GO and the thermally induced phase behavior of PNIPAM. The system can segregate gel phase from aqueous phase for sewage treatments according to temperature increase. M^n+^ indicates metal ions, such as Cu^2+^, Co^2+^, Cd^2+^, Ni^2+^, Pb^2+^, Zn^2+^, and Sr^2+^. BMC, biomolecular condensate; GO, graphene oxide; LCST, *lower* critical solution temperature; PNIPAM, poly(*N*-isopropylacrylamide); UCST, *upper* critical solution temperature.

Phase-separating proteins, especially IDPs, can be typically classified into upper critical solution temperature (UCST) and lower-critical-solution-temperature (LCST) types (78). For proteins with UCST phase behavior, the temperature below a specific threshold initiates phase separation. Conversely, proteins with LCST phase behavior undergoes phase separation at temperatures above the critical temperature (Fig. 4*A*). Notably, the integration of an IDP with UCST behavior and another IDP with LCST behavior can potentially exhibit biphasic behavior (79). This combination employs temperature-sensitive phase behaviors of both UCST and LCST, offering a multifunctional dynamic system in response to temperature changes (80). For instance, synthetic polymers with blocks of polyethylene glycol (LCST) and acrylamide–acrylonitrile copolymers (UCST) can exhibit dual thermoresponsive properties (81).

Jeetain Mittal group elucidated a study for the temperature-controlled phase separation of IDPs (82). By employing a coarse-grained model with a unique amino acid potential sensitive to temperature effects, the research systematically probed and distinguished more than 35 IDPs with different thermoresponsive phase behaviors. The approach offers insights into designing protein sequences with desired thermoresponsive properties and understanding cellular responses to heat stress. Taihong Yan group leveraged a temperature-responsive system for sewage treatment, integrating poly(*N*-isopropyl acrylamide) (PNIPAM) and graphene oxide (GO) (83) (Fig. 4*B*). The GO is enriched in functional groups (carboxyl, hydroxyl, and epoxy) that act as potent metal-chelating agents, whereas PNIPAM, a thermosensitive polymer, switches from an aqueous phase to a gel phase after temperature increase (84). The developed PNIPAM–GO system adsorbs and enriches heavy metal ions into the gel phase when the temperature is above a certain “cloud point.” Thus, this temperature-responsive system utilizes GO's high adsorption capacity and PNIPAM's phase transition characteristics to effectively segregate metal ions from wastewater.

The unique features of temperature-controlled phase separation open up a new avenue for exploring the formation and construction of SBMCs and may have implications for the development of smart biomaterials or biosensors.

### The pH-controlled phase separation

Cellular pH affects protein–protein/RNA interactions, alters protein solubility, and acts as a messenger to transmit stress signals, making it a crucial environmental stimulus for regulating phase separation (85). Phase-separating proteins with pH-dependent properties could initiate phase separation based on the protonation state of their ionizable group. For instance, proteins like elastin-like polypeptides can transition between a soluble state and a phase-separated state in response to changes in pH, making them versatile candidates for pH-responsive biomaterials (86, 87, 88).

The pH is also a vital regulatory stimulus for constructing SBMCs (13, 89). By harnessing the pH-sensitive properties of proteins and polymers, researchers can design responsive systems that effectively operate across various unfriendly conditions, enhancing the functionality and applicability of SBMCs in synthetic biology and bioengineering. Recent studies have demonstrated the utility of pH-responsive systems in drug delivery. Xuesi Chen group reported that a pH-sensitive hydrogel composed of polyacrylic acid can swell or shrink based on environmental pH, thus enabling controlled release of the drugs (90).

## The applications of SBMCs

The phase separation–driven BMCs comprehensively involve spatiotemporal regulation of biological processes, whereas aberrant formations of BMCs are often associated with physiological or pathological disorders (16). The study of *de novo* synthesis of BMCs facilitates the exploration of cellular organization and provides important models for pathogenetic, biomanufacturing, and biosynthetic research (Table 1).

### SBMCs in chromosome structure research

As the largest molecule in cells, chromosome organization is essential for gene expression and genome stability (91, 92). Recently, the crucial roles of phase separation in regulating chromosome structure and organization are increasingly recognized.

**Figure 5:**
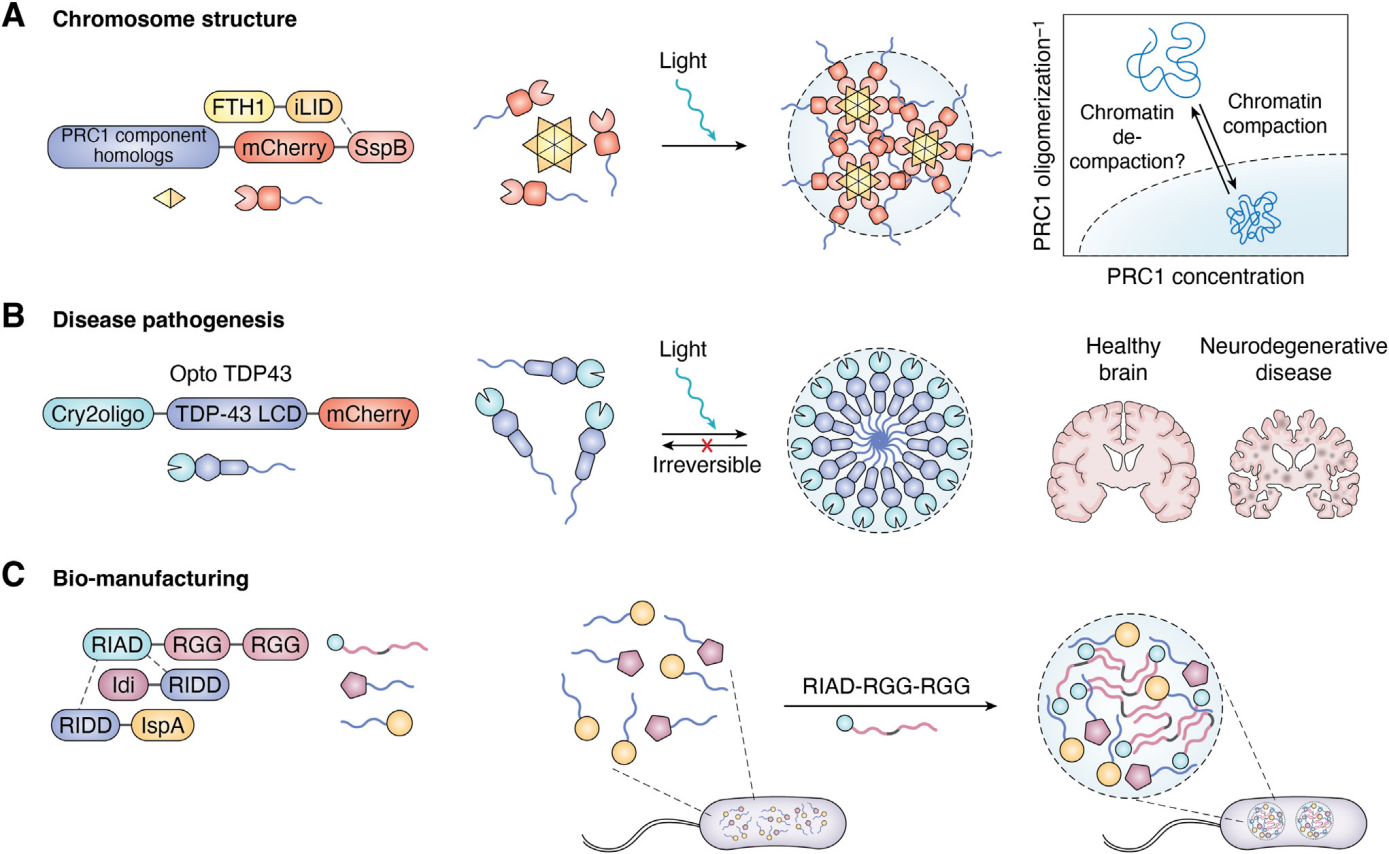
**Applications of SBMCs in biotechnology, biomedicine, and biomanufacturing.***A*, SBMCs in chromosome structure study. A light-activated PRC1 condensate was designed using the Corelets system. One homolog for each canonical subunit of PRC1 is selected and fused with mCherry-SspB individually. The light-activated results demonstrate that only CBX2-Corelets have a significant and sustained effect on chromatin compaction. *B*, SBMCs in neurodegenerative disease. An engineered optoTDP43 system using Cry2oligo-TDP-43-mCherry was constructed. The results indicate that the low-complexity domains (LCDs) of TDP-43 play a central role in promoting stable droplet formation. *C*, SBMCs in biomanufacturing. An SBMC utilizing RGG-RGG scaffold protein and short peptide interaction (RIAD and RIDD) was constructed. The results show that the system efficiently assembles isopentenyl diphosphate isomerase (Idi) and farnesyl pyrophosphate synthase (IspA), leading to improved production of α-farnesene. SBMC, synthesized biomolecular condensate.

Polycomb repressive complex 1 (PRC1) is a chromatin-modifying complex engaged in maintaining the repressed state of chromatin (93). To elucidate the detailed mechanism of PRC1 in chromatin densification, Clifford Brangwynne group designed a photoactivated multicomponent PRC1 condensate *via* the Corelets system (62) (Fig. 5*A*). In this system, one homolog for each canonical subunit of PRC1 is chosen (BMI1, PHC1, CBX2, and RNF2) (94) and individually fused with mCherry-SspB. This configuration allows the controlled formation of condensates, offering a unique observation window for the relationship between PRC1 components and chromatin organization. The findings demonstrate that the condensates formed by CBX2-Corelets enhance chromatin compaction at H3K27me3-rich sites upon light activation. Even after the dissolution of CBX2 condensates, chromatin remains compacted for an extended period, suggesting a sustained effect of condensates on chromatin structure. Conversely, the BMI1-Corelets and PHC1-Corelets did not exhibit any increase in chromatin compaction over time (64). The RNF2-Corelets only exhibited moderate compaction.

Taken together, the development of SBMCs provided a controlled environment for precise regulation of the molecular composition of chromosome structure, offering an ideal platform for chromosome structural research.

### SBMCs in analyzing pathogenesis of neurodegenerative diseases

Extensive research has revealed a significant association between the aberrant formation of BMCs and the onset of neurodegenerative diseases, including amyotrophic lateral sclerosis and frontotemporal dementia (95). Pathological manifestations of these diseases are often characterized by TDP-43 protein lesion accumulation (96).

To comprehend the mechanisms underlying TDP-43 accumulation, Christopher Donnelly conducted a pioneering study using the OptoDroplets system to precisely manipulate the formation of TDP-43 condensates (18, 68) (Fig. 5*B*). By engineering a photogenetic Cry2oligo-TDP-43-mCherry construct (optoTDP43), they successfully induced TDP-43 lesions using targeted blue light stimulation. Their research uncovers that the disease-associated accumulation is mainly facilitated by aberrant interactions of low-complexity domains in TDP-43. Specifically, the opto–low-complexity domains from both wildtype TDP-43 and amyotrophic lateral sclerosis–related mutations accelerate the formation of stable droplets during phase separation. Pathological features associated with droplet contents were identified in this system, including excessive phosphorylation and positive expression of the aggrephagy-related protein p62 (97).

The study highlighted SBMCs' critical function in analyzing abnormal phase separation of TDP-43, indicating that they function as a useful model in studying the pathogenesis of neurodegenerative disorders.

### SBMCs in biomanufacturing study

In biocatalytic systems, BMCs facilitate precise control of reaction environments and substrate selectivity (98). Moreover, the condensation of catalytic enzymes by phase separation streamlines metabolic flows and enhances metabolite yields, enabling engineering the BMCs for biomanufacturing (99) (Table 1).

Jiang Xia group developed a multienzyme-based SBMC facilitating the synthesis of the natural compound of α-farnesene (100). The construction is based on the scaffold RGG–RGG protein and utilizes a pair of short peptide interactions (RIAD and RIDD) (101) to assemble isopentenyl diphosphate isomerase (Idi) and farnesyl pyrophosphate synthase (IspA). The designed fusion proteins Idi-RIDD and RIDD-IspA can be recruited by the RIAD–RGG–RGG condensates. Their findings show that the multienzyme-based SBMCs enhanced the biosynthesis of α-farnesene and improved the reaction efficiency and product purity in *E*. *coli* (Fig. 5*C*).

Recently, the Xiaoxia Xia group introduced another SBMC to produce 1,3-diaminopropane (1,3-DAP) using spider-silk-protein–based condensates (15). The α-ketoglutarate 4-aminotransferase (Dat) and ւ-2,4-diaminobutyrate decarboxylase (Ddc) are known to be responsible for the conversion of aspartate β-semialdehyde into 1,3-DAP. Xiaoxia Xia *et al.* designed fusion proteins of Dat and Ddc with the spider silk protein I16, respectively. Coexpression of the fusion proteins resulted in the emergence of condensates within recombinant *E. coli* cells, yielding a slightly higher 1,3-DAP level than that from the original cells.

Together, these studies demonstrated the construction of SBMCs in living cells and their functions in engineering biosynthesis, providing new ideas for the application of phase separation in biomanufacturing.

### SBMCs in artificial cell design and drug delivery

Phase separation refers to the process by which a homogeneous mixture segregates into distinct phases with varying concentrations of biomolecules. In the development of cellular mimics, SBMCs are particularly valued for their ability to form encapsulated compartments while maintaining relatively high permeability, which facilitates molecular exchange and functional activity (102). Recent studies have demonstrated that the phase-separated artificial cell systems can efficiently support cellular organization, signal processing, and metabolic engineering (103, 104). The Yan Qiao group introduced phase separation–based artificial organelles by integrating membraneless coacervate microdroplets into a proteinosome. These hierarchical protocells effectively enrich biomolecular reactants within confined organelles, thereby accelerating enzymatic reactions. Furthermore, these protocells can detect extracellular signals, respond to stimuli, and enable biochemical logic gates, providing a versatile platform for studying cellular organization, complex metabolic networks, and chemical computation (105). Subsequently, the Yan Qiao group developed artificial multicellular networks based on a binary population of coacervate microdroplets. These networks facilitate macromolecular self-sorting, spatially localized biocatalysis, and interdroplet molecular translocation, paving the way for the synthesis of multicomponent protocells and the creation of artificial tissues with collective functions (106).

Meanwhile, SBMCs have emerged as powerful tools for drug delivery in recent years, owing to their unique ability to enhance the retention of small molecules (107). The Zhen Gu group developed an innovative strategy for the *in situ* formation of these condensates as drug reservoirs within cancer cells. By utilizing positively charged histones and doxorubicin-intercalated DNA strands linked *via* a click-to-release reaction, their approach induces phase separation and leads to the formation of large BMCs that effectively retain chemotherapeutic agents. This novel method shows promise for improving cancer treatment efficacy, particularly in overcoming drug-resistant tumors (108). Similarly, the Aleksandar Matic group devised a formulation strategy for controlled drug release using phase-separated polymer blends in solid oral dosage forms. By harnessing the phase separation between hydrophobic and hydrophilic polymers, they create a porous network upon dissolution. Imaging techniques reveal how polymer composition and drug presence influence morphology and drug distribution, highlighting the potential of tailoring polymer matrix morphology for precise control over drug release in oral formulations (109).

In summary, the integration of SBMCs into artificial cell design and drug delivery represents significant advancements in synthetic biology and therapeutic innovation. These systems hold the potentials for advancing cell synthesis and enabling precision medicine in the future.

## Challenges and prospects

The conceptualization, construction, and application of SBMCs is at the forefront of cell biology and synthetic biology. In the current review, we elucidate the core principles of phase separation, especially emphasizing the roles of multivalent interactions in driving it. The current methodologies in attaining spatiotemporally controlled SBMCs are reviewed, encompassing regulatory strategies ranging from light, chemical, enzyme, and temperature-controlled phase separations. With the development of these techniques/systems, the recent applications of SBMCs in biotechnology, biomedicine, and biomanufacturing are introduced.

Although current application cases are still limited, the phase separation–driven SBMCs are increasingly recognized as a new research paradigm for cell organization and metabolic engineering (110, 111). Meanwhile, more challenges in this field require attention. One of the most challenges is maintaining the liquid structures of SBMCs. The SBMCs must ensure a delicate balance to prevent unnecessary aggregation or dispersal. Any disturbances to this balance could jeopardize the structural integrity and functionality of organelles, and especially, is implicated in disease progression (112). Future directions should focus on unraveling the dynamic structures of BMCs, using synthetic biology, mathematical modeling, and cutting-edge structural biology to offer a deeper understanding of their organization and aging mechanisms (113).

Another challenge is the spatiotemporal regulation of SBMCs. The precise regulation of SBMCs enables the membraneless organelles to perform their unique functions right in place and time (114). However, as outlined previously, the regulatory methods characterized as sensitive, reversible, and practical are still limited (1, 16, 115). Studies need to focus on how phase separation is coordinated and regulated sophisticatedly, both *in vitro* and in natural living cells.

The other challenge lies in the potential interactions between SBMCs and the endogenous structures of cells, such as the cellular membrane, chromosomes, and endoplasmic reticulum. These interactions profoundly influence the formation, stability, and functionality of both SBMCs and the endogenous structures themselves (116). The SBMCs may associate with membranes to form membrane-linked phase-separated entities, which play crucial roles in cellular signaling and material transport (117). Further investigation into these interactions could offer valuable insights for the design and application of these SBMCs.

The last challenge is the achievement of functionalized SBMCs. Design interactions between the condensate components and signaling/catalytic proteins are of great importance to functional organelles (114). However, multiple interactions will significantly increase the operational difficulty of SBMCs and influence their structures and efficiencies (58). Due to the understanding of the interaction specificity of natural BMCs being incomplete, future directions should include the orthogonal design of interaction circuits and, importantly, answer where the binding specificity of BMCs arises from.

In summary, the comprehensive understanding and application of SBMCs will illuminate our future work, enriching our knowledge of chromosome structure, cellular metabolism, and neurodegenerative diseases, and promoting the development of novel biosensors and therapeutic strategies.

## Conflicts of interest

The authors declare that they have no conflicts of interest with the contents of this article.

## References

[1] Banani S.F., Lee H.O., Hyman A.A., Rosen M.K. (2017). Biomolecular condensates: organizers of cellular biochemistry. Nat. Rev. Mol. Cell Biol..

[2] Gomes E., Shorter J. (2019). The molecular language of membraneless organelles. J. Biol. Chem..

[3] Hirose T., Ninomiya K., Nakagawa S., Yamazaki T. (2023). A guide to membraneless organelles and their various roles in gene regulation. Nat. Rev. Mol. Cell Biol..

[4] Brangwynne C.P., Tompa P., Pappu R.V. (2015). Polymer physics of intracellular phase transitions. Nat. Phys..

[5] Wu J., Chen B., Liu Y., Ma L., Huang W., Lin Y. (2022). Modulating gene regulation function by chemically controlled transcription factor clustering. Nat. Commun..

[6] Schneider N., Wieland F.-G., Kong D., Fischer A.A.M., Hörner M., Timmer J. (2021). Liquid-liquid phase separation of light-inducible transcription factors increases transcription activation in mammalian cells and mice. Sci. Adv..

[7] Strom A.R., Emelyanov A.V., Mir M., Fyodorov D.V., Darzacq X., Karpen G.H. (2017). Phase separation drives heterochromatin domain formation. Nature.

[8] Pessina F., Gioia U., Brandi O., Farina S., Ceccon M., Francia S. (2021). DNA damage triggers a new phase in neurodegeneration. Trends Genet..

[9] Tan W., Cheng S., Li Y., Li X.-Y., Lu N., Sun J. (2022). Phase separation modulates the assembly and dynamics of a polarity-related scaffold-signaling hub. Nat. Commun..

[10] Liu Z., Yang Y., Gu A., Xu J., Mao Y., Lu H. (2020). Par complex cluster formation mediated by phase separation. Nat. Commun..

[11] Nozawa R., Yamamoto T., Takahashi M., Tachiwana H., Maruyama R., Hirota T. (2020). Nuclear microenvironment in cancer: control through liquid-liquid phase separation. Cancer Sci..

[12] Zhang H., Ji X., Li P., Liu C., Lou J., Wang Z. (2020). Liquid-liquid phase separation in biology: mechanisms, physiological functions and human diseases. Sci. China Life Sci..

[13] Jin X., Zhou M., Chen S., Li D., Cao X., Liu B. (2022). Effects of pH alterations on stress- and aging-induced protein phase separation. Cell. Mol. Life Sci..

[14] Hilditch A.T., Romanyuk A., Cross S.J., Obexer R., McManus J.J., Woolfson D.N. (2024). Assembling membraneless organelles from de novo designed proteins. Nat. Chem..

[15] Wei S.-P., Qian Z.-G., Hu C.-F., Pan F., Chen M.-T., Lee S.Y. (2020). Formation and functionalization of membraneless compartments in Escherichia coli. Nat. Chem. Biol..

[16] Mehta S., Zhang J. (2022). Liquid–liquid phase separation drives cellular function and dysfunction in cancer. Nat. Rev. Cancer.

[17] Zhao P., Han W., Shu Y., Li M., Sun Y., Sui X. (2023). Liquid–liquid phase separation drug aggregate: merit for oral delivery of amorphous solid dispersions. J. Control. Release.

[18] Mann J.R., Gleixner A.M., Mauna J.C., Gomes E., DeChellis-Marks M.R., Needham P.G. (2019). RNA binding antagonizes neurotoxic phase transitions of TDP-43. Neuron.

[19] Wang Y., Yu C., Pei G., Jia W., Li T., Li P. (2023). Dissolution of oncofusion transcription factor condensates for cancer therapy. Nat. Chem. Biol..

[20] Li P., Banjade S., Cheng H.-C., Kim S., Chen B., Guo L. (2012). Phase transitions in the assembly of multivalent signalling proteins. Nature.

[21] Lotthammer J.M., Ginell G.M., Griffith D., Emenecker R.J., Holehouse A.S. (2024). Direct prediction of intrinsically disordered protein conformational properties from sequence. Nat. Methods.

[22] Agarwal A., Arora L., Rai S.K., Avni A., Mukhopadhyay S. (2022). Spatiotemporal modulations in heterotypic condensates of prion and α-synuclein control phase transitions and amyloid conversion. Nat. Commun..

[23] Peng P.-H., Hsu K.-W., Wu K.-J. (2021). Liquid-liquid phase separation (LLPS) in cellular physiology and tumor biology. Am. J. Cancer Res..

[24] Luo Y., Na Z., Slavoff S.A. (2018). P-bodies: composition, properties, and functions. Biochemistry.

[25] Schmidt H.B., Barreau A., Rohatgi R. (2019). Phase separation-deficient TDP43 remains functional in splicing. Nat. Commun..

[26] Mohanty P., Kapoor U., Sundaravadivelu Devarajan D., Phan T.M., Rizuan A., Mittal J. (2022). Principles governing the phase separation of multidomain proteins. Biochemistry.

[27] Pappu R.V., Cohen S.R., Dar F., Farag M., Kar M. (2023). Phase transitions of associative biomacromolecules. Chem. Rev..

[28] Vernon R.M., Chong P.A., Tsang B., Kim T.H., Bah A., Farber P. (2018). Pi-Pi contacts are an overlooked protein feature relevant to phase separation. eLife.

[29] Martin E.W., Holehouse A.S., Peran I., Farag M., Incicco J.J., Bremer A. (2020). Valence and patterning of aromatic residues determine the phase behavior of prion-like domains. Science.

[30] Li H.-R., Chiang W.-C., Chou P.-C., Wang W.-J., Huang J. (2018). TAR DNA-binding protein 43 (TDP-43) liquid–liquid phase separation is mediated by just a few aromatic residues. J. Biol. Chem..

[31] Wang J., Choi J.-M., Holehouse A.S., Lee H.O., Zhang X., Jahnel M. (2018). A molecular grammar governing the driving forces for phase separation of prion-like RNA binding proteins. Cell.

[32] Lin Y., Currie S.L., Rosen M.K. (2017). Intrinsically disordered sequences enable modulation of protein phase separation through distributed tyrosine motifs. J. Biol. Chem..

[33] Xiang S., Kato M., Wu L.C., Lin Y., Ding M., Zhang Y. (2015). The LC domain of hnRNPA2 adopts similar conformations in hydrogel polymers, liquid-like droplets, and nuclei. Cell.

[34] Krainer G., Welsh T.J., Joseph J.A., Espinosa J.R., Wittmann S., De Csilléry E. (2021). Reentrant liquid condensate phase of proteins is stabilized by hydrophobic and non-ionic interactions. Nat. Commun..

[35] Dooam P., Hankyul L., Hyungjun K., JeongMo C. (2022). Thermodynamics of π–π interactions of Benzene and Phenol in Water. Int. J. Mol. Sci..

[36] Wright P.E., Dyson H.J. (2015). Intrinsically disordered proteins in cellular signaling and regulation. Nat. Rev. Mol. Cel. Biol..

[37] Hong Y., Najafi S., Casey T., Shea J.-E., Han S.-I., Hwang D.S. (2022). Hydrophobicity of arginine leads to reentrant liquid-liquid phase separation behaviors of arginine-rich proteins. Nat. Commun..

[38] Murray D.T., Kato M., Lin Y., Thurber K.R., Hung I., McKnight S.L. (2017). Structure of FUS protein fibrils and its relevance to self-assembly and phase separation of low-complexity domains. Cell.

[39] Abeln S., Vendruscolo M., Dobson C.M., Frenkel D. (2014). A simple lattice model that captures protein folding, aggregation and amyloid formation. PLoS One.

[40] Vidal Ceballos A., Díaz A J.A., Preston J.M., Vairamon C., Shen C., Koder R.L. (2022). Liquid to solid transition of elastin condensates. Proc. Natl. Acad. Sci. U. S. A..

[41] Garabedian M.V., Wang W., Dabdoub J.B., Tong M., Caldwell R.M., Benman W. (2021). Designer membraneless organelles sequester native factors for control of cell behavior. Nat. Chem. Biol..

[42] Chong P.A., Vernon R.M., Forman-Kay J.D. (2018). RGG/RG motif regions in RNA binding and phase separation. J. Mol. Biol..

[43] Quiroz F.G., Chilkoti A. (2015). Sequence heuristics to encode phase behaviour in intrinsically disordered protein polymers. Nat. Mater..

[44] Budini M., Buratti E., Stuani C., Guarnaccia C., Romano V., De Conti L. (2012). Cellular model of TAR DNA-binding protein 43 (TDP-43) aggregation based on its C-terminal Gln/Asn-rich region. J. Biol. Chem..

[45] Osherovich L.Z., Weissman J.S. (2001). Multiple Gln/Asn-rich prion domains confer susceptibility to induction of the yeast [PSI] prion. Cell.

[46] Dignon G.L., Zheng W., Kim Y.C., Best R.B., Mittal J. (2018). Sequence determinants of protein phase behavior from a coarse-grained model. PLoS Comput. Biol..

[47] Qian D., Michaels T.C.T., Knowles T.P.J. (2022). Analytical solution to the Flory–Huggins model. J. Phys. Chem. Lett..

[48] Huggins M.L. (1941). Solutions of long chain compounds. J. Chem. Phys..

[49] Flory P.J. (1942). Thermodynamics of high polymer solutions. J. Chem. Phys..

[50] Choi J.-M., Dar F., Pappu R.V. (2019). LASSI: a lattice model for simulating phase transitions of multivalent proteins. PLoS Comput. Biol..

[51] Nott T.J., Petsalaki E., Farber P., Jervis D., Fussner E., Plochowietz A. (2015). Phase transition of a disordered nuage protein generates environmentally responsive membraneless organelles. Mol. Cell.

[52] Schmidt H.B., Görlich D. (2015). Nup98 FG domains from diverse species spontaneously phase-separate into particles with nuclear pore-like permselectivity. eLife.

[53] Gui X., Luo F., Li Y., Zhou H., Qin Z., Liu Z. (2019). Structural basis for reversible amyloids of hnRNPA1 elucidates their role in stress granule assembly. Nat. Commun..

[54] Alberti S., Gladfelter A., Mittag T. (2019). Considerations and challenges in studying liquid-liquid phase separation and biomolecular condensates. Cell.

[55] Niopek D., Benzinger D., Roensch J., Draebing T., Wehler P., Eils R. (2014). Engineering light-inducible nuclear localization signals for precise spatiotemporal control of protein dynamics in living cells. Nat. Commun..

[56] Bugaj L.J., Choksi A.T., Mesuda C.K., Kane R.S., Schaffer D.V. (2013). Optogenetic protein clustering and signaling activation in mammalian cells. Nat. Methods.

[57] Strickland D., Lin Y., Wagner E., Hope C.M., Zayner J., Antoniou C. (2012). TULIPs: tunable, light-controlled interacting protein tags for cell biology. Nat. Methods.

[58] Shin Y., Berry J., Pannucci N., Haataja M.P., Toettcher J.E., Brangwynne C.P. (2017). Spatiotemporal control of intracellular phase transitions using light-activated optoDroplets. Cell.

[59] Kaberniuk A.A., Shemetov A.A., Verkhusha V.V. (2016). A bacterial phytochrome-based optogenetic system controllable with near-infrared light. Nat. Methods.

[60] Milbradt A.G., Kulkarni M., Yi T., Takeuchi K., Sun Z.-Y.J., Luna R.E. (2011). Structure of the VP16 transactivator target in the Mediator. Nat. Struct. Mol. Biol..

[61] Gossen M., Bujard H. (1992). Tight control of gene expression in mammalian cells by tetracycline-responsive promoters. Proc. Natl. Acad. Sci. U. S. A..

[62] Bracha D., Walls M.T., Wei M.-T., Zhu L., Kurian M., Avalos J.L. (2018). Mapping local and global liquid phase behavior in living cells using photo-oligomerizable seeds. Cell.

[63] Shin Y., Chang Y.-C., Lee D.S.W., Berry J., Sanders D.W., Ronceray P. (2018). Liquid nuclear condensates mechanically sense and restructure the genome. Cell.

[64] Shimobayashi S.F., Ronceray P., Sanders D.W., Haataja M.P., Brangwynne C.P. (2021). Nucleation landscape of biomolecular condensates. Nature.

[65] Dine E., Gil A.A., Uribe G., Brangwynne C.P., Toettcher J.E. (2018). Protein phase separation provides long-term memory of transient spatial stimuli. Cell Syst..

[66] Ren S., Sato R., Hasegawa K., Ohta H., Masuda S. (2013). A predicted structure for the PixD–PixE complex determined by homology modeling, docking simulations, and a mutagenesis study. Biochemistry.

[67] Li M., Park B.M., Dai X., Xu Y., Huang J., Sun F. (2022). Controlling synthetic membraneless organelles by a red-light-dependent singlet oxygen-generating protein. Nat. Commun..

[68] Taslimi A., Vrana J.D., Chen D., Borinskaya S., Mayer B.J., Kennedy M.J. (2014). An optimized optogenetic clustering tool for probing protein interaction and function. Nat. Commun..

[69] Nakamura H., Lee A.A., Afshar A.S., Watanabe S., Rho E., Razavi S. (2018). Intracellular production of hydrogels and synthetic RNA granules by multivalent molecular interactions. Nat. Mater..

[70] Wu H.D., Kikuchi M., Dagliyan O., Aragaki A.K., Nakamura H., Dokholyan N.V. (2020). Rational design and implementation of a chemically inducible heterotrimerization system. Nat. Methods.

[71] Hong K., Song D., Jung Y. (2020). Behavior control of membrane-less protein liquid condensates with metal ion-induced phase separation. Nat. Commun..

[72] Babinchak W.M., Dumm B.K., Venus S., Boyko S., Putnam A.A., Jankowsky E. (2020). Small molecules as potent biphasic modulators of protein liquid-liquid phase separation. Nat. Commun..

[73] Younan N.D., Viles J.H. (2015). A comparison of three fluorophores for the detection of amyloid fibers and prefibrillar oligomeric assemblies. ThT (thioflavin T); ANS (1-anilinonaphthalene-8-sulfonic acid); and bisANS (4,4′-dianilino-1,1′-binaphthyl-5,5′-disulfonic acid). Biochemistry.

[74] Schuster B.S., Reed E.H., Parthasarathy R., Jahnke C.N., Caldwell R.M., Bermudez J.G. (2018). Controllable protein phase separation and modular recruitment to form responsive membraneless organelles. Nat. Commun..

[75] Burke K.A., Janke A.M., Rhine C.L., Fawzi N.L. (2015). Residue-by-residue view of in vitro FUS granules that bind the C-terminal domain of RNA polymerase II. Mol. Cell.

[76] Abdelkader E.H., Otting G. (2021). NT∗-HRV3CP: an optimized construct of human rhinovirus 14 3C protease for high-yield expression and fast affinity-tag cleavage. J. Biotechnol..

[77] Cesaratto F., Burrone O.R., Petris G. (2016). Tobacco Etch Virus protease: a shortcut across biotechnologies. J. Biotechnol..

[78] Ruff K.M., Roberts S., Chilkoti A., Pappu R.V. (2018). Advances in understanding stimulus-responsive phase behavior of intrinsically disordered protein polymers. J. Mol. Biol..

[79] Dai Y., You L., Chilkoti A. (2023). Engineering synthetic biomolecular condensates. Nat. Rev. Bioeng..

[80] Forero-Martinez N.C., Cortes-Huerto R., Benedetto A., Ballone P. (2022). Thermoresponsive ionic liquid/water mixtures: from nanostructuring to phase separation. Molecules.

[81] Dong Z., Mao J., Wang D., Yang M., Ji X. (2015). Synthesis and multi-stimuli-responsive behavior of poly(*N* , *N* -dimethylaminoethyl methacrylate) spherical brushes under different modes of confinement in solution. Langmuir.

[82] Dignon G.L., Zheng W., Kim Y.C., Mittal J. (2019). Temperature-controlled liquid–liquid phase separation of disordered proteins. ACS Cent. Sci..

[83] Wang W., Liu Z., Wang R., Cao M., Chen Y., Lu X. (2023). A novel strategy for efficient removal of hazardous metal ions based on thermoresponsive phase separation of the PNIPAM/GO system. Chem. Eng. J..

[84] Cao M., Wang Y., Hu X., Gong H., Li R., Cox H. (2019). Reversible thermoresponsive peptide–PNIPAM hydrogels for controlled drug delivery. Biomacromolecules.

[85] Sun Y., Lau S.Y., Lim Z.W., Chang S.C., Ghadessy F., Partridge A. (2022). Phase-separating peptides for direct cytosolic delivery and redox-activated release of macromolecular therapeutics. Nat. Chem..

[86] Roberts S., Dzuricky M., Chilkoti A. (2015). Elastin-like polypeptides as models of intrinsically disordered proteins. FEBS Lett..

[87] Girotti A., Reguera J., Arias F.J., Alonso M., Testera A.M., Rodríguez-Cabello J.C. (2004). Influence of the molecular weight on the inverse temperature transition of a model genetically engineered elastin-like pH-responsive polymer. Macromolecules.

[88] Dumetz A.C., Chockla A.M., Kaler E.W., Lenhoff A.M. (2008). Effects of pH on protein–protein interactions and implications for protein phase behavior. Biochim. Biophys. Acta.

[89] Adame-Arana O., Weber C.A., Zaburdaev V., Prost J., Jülicher F. (2020). Liquid phase separation controlled by pH. Biophys. J..

[90] Gao X., He C., Xiao C., Zhuang X., Chen X. (2013). Biodegradable pH-responsive polyacrylic acid derivative hydrogels with tunable swelling behavior for oral delivery of insulin. Polymer.

[91] Yu M., Ren B. (2017). The three-dimensional organization of mammalian genomes. Annu. Rev. Cell Dev. Biol..

[92] Pombo A., Dillon N. (2015). Three-dimensional genome architecture: players and mechanisms. Nat. Rev. Mol. Cell Biol..

[93] Shukla S., Ying W., Gray F., Yao Y., Simes M.L., Zhao Q. (2021). Small molecule inhibitors targeting Polycomb repressive complex 1 RING domain. Nat. Chem. Biol..

[94] Eeftens J.M., Kapoor M., Michieletto D., Brangwynne C.P. (2021). Polycomb condensates can promote epigenetic marks but are not required for sustained chromatin compaction. Nat. Commun..

[95] Mensah M.A., Niskanen H., Magalhaes A.P., Basu S., Kircher M., Sczakiel H.L. (2023). Aberrant phase separation and nucleolar dysfunction in rare genetic diseases. Nature.

[96] Ling S.-C., Polymenidou M., Cleveland D.W. (2013). Converging mechanisms in ALS and FTD: disrupted RNA and protein homeostasis. Neuron.

[97] Kurashige T., Kuramochi M., Ohsawa R., Yamashita Y., Shioi G., Morino H. (2021). Optineurin defects cause TDP43-pathology with autophagic vacuolar formation. Neurobiol. Dis..

[98] Sweetlove L.J., Fernie A.R. (2018). The role of dynamic enzyme assemblies and substrate channelling in metabolic regulation. Nat. Commun..

[99] Lim S., Clark D.S. (2024). Phase-separated biomolecular condensates for biocatalysis. Trends Biotechnol..

[100] Wang Y., Liu M., Wei Q., Wu W., He Y., Gao J. (2022). Phase-separated multienzyme compartmentalization for terpene biosynthesis in a prokaryote. Angew. Chem. Int. Ed..

[101] Purwani N.N., Martin C., Savino S., Fraaije M.W. (2021). Modular assembly of phosphite dehydrogenase and phenylacetone monooxygenase for tuning cofactor regeneration. Biomolecules.

[102] Nag N., Sasidharan S., Uversky V.N., Saudagar P., Tripathi T. (2022). Phase separation of FG-nucleoporins in nuclear pore complexes. Biochim Biophys Acta Mol Cell Res.

[103] Engelhart A.E., Adamala K.P., Szostak J.W. (2016). A simple physical mechanism enables homeostasis in primitive cells. Nat. Chem..

[104] Adamala K.P., Martin-Alarcon D.A., Guthrie-Honea K.R., Boyden E.S. (2017). Engineering genetic circuit interactions within and between synthetic minimal cells. Nat. Chem..

[105] Mu W., Ji Z., Zhou M., Wu J., Lin Y., Qiao Y. (2021). Membrane-confined liquid-liquid phase separation toward artificial organelles. Sci. Adv..

[106] Mu W., Jia L., Zhou M., Wu J., Lin Y., Mann S. (2024). Superstructural ordering in self-sorting coacervate-based protocell networks. Nat. Chem..

[107] Conti B.A., Oppikofer M. (2022). Biomolecular condensates: new opportunities for drug discovery and RNA therapeutics. Trends Pharmacol. Sci..

[108] Liang T., Dong Y., Cheng I., Wen P., Li F., Liu F. (2024). In situ formation of biomolecular condensates as intracellular drug reservoirs for augmenting chemotherapy. Nat. Biomed. Eng..

[109] Olsson M., Storm R., Björn L., Lilja V., Krupnik L., Chen Y. (2024). Phase-separated polymer blends for controlled drug delivery by tuning morphology. Commun. Mater..

[110] Wang B., Zhang L., Dai T., Qin Z., Lu H., Zhang L. (2021). Liquid–liquid phase separation in human health and diseases. Signal Transduct Target Ther..

[111] Jin X., Lee J.E., Schaefer C., Luo X., Wollman A.J.M., Payne-Dwyer A.L. (2021). Membraneless organelles formed by liquid-liquid phase separation increase bacterial fitness. Sci. Adv..

[112] Lin Y., Protter D.S.W., Rosen M.K., Parker R. (2015). Formation and maturation of phase-separated liquid droplets by RNA-binding proteins. Mol. Cell.

[113] Peran I., Mittag T. (2020). Molecular structure in biomolecular condensates. Curr. Opin. Struct. Biol..

[114] Banani S.F., Rice A.M., Peeples W.B., Lin Y., Jain S., Parker R. (2016). Compositional control of phase-separated cellular bodies. Cell.

[115] Dignon G.L., Best R.B., Mittal J. (2020). Biomolecular phase separation: from molecular driving forces to macroscopic properties. Annu. Rev. Phys. Chem..

[116] Kim N., Yun H., Lee H., Yoo J.-Y. (2024). Interplay between membranes and biomolecular condensates in the regulation of membrane-associated cellular processes. Exp. Mol. Med..

[117] Zhao Y.G., Zhang H. (2020). Phase separation in membrane biology: the interplay between membrane-bound organelles and membraneless condensates. Dev. Cell.

[118] Zhao E.M., Suek N., Wilson M.Z., Dine E., Pannucci N.L., Gitai Z. (2019). Light-based control of metabolic flux through assembly of synthetic organelles. Nat. Chem. Biol..

[119] Wee W.A., Sugiyama H., Park S. (2021). Photoswitchable single-stranded DNA-peptide coacervate formation as a dynamic system for reaction control. iScience.

[120] Liu J., Zhorabek F., Dai X., Huang J., Chau Y. (2022). Minimalist design of an intrinsically disordered protein-mimicking scaffold for an artificial membraneless organelle. ACS Cent. Sci..

[121] Dzuricky M., Rogers B.A., Shahid A., Cremer P.S., Chilkoti A. (2020). De novo engineering of intracellular condensates using artificial disordered proteins. Nat. Chem..

[122] Zhou P., Liu H., Meng X., Zuo H., Qi M., Guo L. (2023). Engineered artificial membraneless organelles in Saccharomyces cerevisiae to enhance chemical production. Angew. Chem. Int. Ed..

[123] Li J., Yang C., Zhang L., Li C., Xie S., Fu T. (2023). Phase separation of DNA-encoded artificial cells boosts signal amplification for biosensing. Angew. Chem. Int. Ed..

